# A comprehensive county level model to identify factors affecting hospital capacity and predict future hospital demand

**DOI:** 10.1038/s41598-021-02376-y

**Published:** 2021-11-29

**Authors:** Tanmoy Bhowmik, Naveen Eluru

**Affiliations:** Department of Civil, Environmental and Construction Engineering, University of Central Florida, Orlando, USA

**Keywords:** Health care, Health policy, Health services, Public health

## Abstract

The sustained COVID-19 case numbers and the associated hospitalizations have placed a substantial burden on health care ecosystem comprising of hospitals, clinics, doctors and nurses. However, as of today, only a small number of studies have examined detailed hospitalization data from a planning perspective. The current study develops a comprehensive framework for understanding the critical factors associated with county level hospitalization and ICU usage rates across the US employing a host of independent variables. Drawing from the recently released Department of Health and Human Services weekly hospitalization data, we study the overall hospitalization and ICU usage—not only COVID-19 hospitalizations. Developing a framework that examines overall hospitalizations and ICU usage can better reflect the plausible hospital system recovery path to pre-COVID level hospitalization trends. The models are subsequently employed to generate predictions for county level hospitalization and ICU usage rates in the future under several COVID-19 transmission scenarios considering the emergence of new COVID-19 variants and vaccination rates. The exercise allows us to identify vulnerable counties and regions under stress with high hospitalization and ICU rates that can be assisted with remedial measures. Further, the model will allow hospitals to understand evolving displaced non-COVID hospital demand.

## Introduction

The Corona Virus Disease (COVID-19) continues to significantly burden social, economic and health systems across the world. As of February 2021, the number of confirmed cases in the world have surpassed 100 million while US alone accounts for about a quarter of these cases^1^. In fact, nearly half of the 28 million cases were reported in the past three months. The total fatalities in the US associated with COVID-19 have crossed 500,000 while daily deaths have averaged nearly 4000 in recent weeks^2^. Further, hospitalization rates have experienced a staggering rise with a peak of about 132 thousand COVID patients hospitalized^3^. Intensive care units (ICUs) are also alarmingly short on space with nearly 33% of the ICUs at a more than 80% occupancy^5^ and a subset of these ICUs at nearly full occupancy. It is an understatement to suggest that the sustained COVID-19 case numbers and the associated hospitalizations have placed a substantial burden on health care ecosystem comprising of hospitals, clinics, doctors and nurses. The hospital systems have been overwhelmed with COVID-19 cases contributing in the range of 20–50% of the hospitalizations at various facilities. An oft neglected aspect of COVID-19 impacts includes members of the community not contracting COVID-19 but being affected by it. For example, the increasing COVID-19 cases has potentially affected non-COVID hospitalizations i.e. patients are either delaying elective procedures or are being forced to reschedule due to lack of availability in hospitals in some regions. A study conducted in UK^6^ already anticipated an increase in cancer deaths due to the reduced cancer service triggered by the evolving COVID-19 pandemic. The effect of these delays are likely to extend for months (if not years) after COVID-19 cases are brought under control.

The emergency use authorization of two vaccines (Pfizer-BioNTech and Moderna) offers a potential path to bringing the virus under control. While the initial vaccination rates in the US were slower than anticipated, in recent days, a vaccination rate above 1 million shots per day has been regularly achieved. Even with this impressive vaccination rate, achieving herd immunity could potentially happen only in the early fall months. Further, as new variants of COVID-19 arise with significantly higher transmissibility^7^, there is a need to maintain our guard in monitoring the cases and resulting consequences (hospitalizations and fatalities). Under these circumstances, there is a need to examine hospitalization and ICU bed usage rates for two reasons. *First*, to ensure there are adequate hospital facilities for COVID-19 patients until we significantly reduce the threat of the virus. *Second*, an understanding of hospitalization trends over time will allow us to examine the time when hospital systems can revert to pre-COVID demand. The stabilization of the hospital system will only occur after the displaced health needs of non- COVID patients (delayed surgeries and treatments) are addressed. Towards addressing these aforementioned challenges, the current study develops a comprehensive framework for understanding the critical factors associated with county level hospitalization and ICU usage rates across the US. We estimate the overall hospitalization and ICU usage as two components: (1) COVID-19 hospitalization and ICU per capita rates and (2) non-COVID hospitalization and ICU per capita rates. The consideration of two components (as opposed to the total rate) will allow us to recognize the distinct impact of various factors on each component. The estimated models are employed to generate predictions for hospitalization and ICU usage rates into the future under a host of COVID-19 transmission scenarios considering the new variants of COVID-19 and vaccination impacts.

A significant amount of research has been conducted on understanding COVID-19 transmission (see^8,9^ for details). However, only a limited amount of research has examined detailed hospitalization data^10–19^, particularly from a public health planning perspective^13,14,16,19^. At the planning level, the studies considered the number of people admitted to hospital and ICUs due to COVID-19 as the response variable and employed either time series^14,16^ or online interactive models^13,19^ for their analysis. In terms of spatial resolution, different spatial resolutions are explored including region^14^, county^19^, country^13,16^ and city^13^. With respect to independent variables, the studies found different factors affecting COVID hospitalization and ICU usage rates including demographics (particularly age and racial distribution^13,19^), mobility trends^14,19^ and COVID-19 transmission rates^14,16^. Earlier research efforts on COVID hospitalizations offered several insights. However, given the evolving challenges of COVID-19 the research efforts are still in their infancy.

The current research contributes to the burgeoning literature on COVID-19 hospitalization with the following objectives. *First*, national level hospitalization data with COVID-19 hospitalization rates at fine spatial resolution have not been easily available. A fine resolution national dataset can allow us to draw insights on the difference across communities that are severely affected relative to other communities. The current research draws on the recently released Department of Health and Human Services (DHHS) weekly hospitalization data for our analysis. Further, the data employed covers hospitalization data during the peak of the pandemic—from August 28th, 2020 to January 22nd, 2021- with 21 weeks of data across each county.

*Second*, the current study focuses on understanding the influence of COVID-19 on overall hospitalizations—not only COVID-19 hospitalizations. We recognize that the impact of COVID-19 on overall hospitalizations is going to persist even after the pandemic ends. The displaced hospital demand during the pandemic will need to be addressed once COVID-19 cases reduce substantially. Thus, developing a framework that examines overall hospitalizations and ICU bed usage as a result of COVID and non-COVID hospitalizations can better reflect the plausible hospital system recovery path to pre-COVID level hospitalization trends.

*Third*, earlier work on hospitalizations employed simple time series models or focused on descriptive and visualization exercises to understand the association between hospitalization and other factors. In our study, we employ a robust modeling framework to analyze the hospitalization and ICU usage demand at a county level. Specifically, we adopt a mixed linear modelling approach^8^ to account for the repeated observations at the county level (multiple weeks of data). Further, the model estimation exercise is carried out using a comprehensive list of county level independent variables including (a) COVID-19 transmission related factors; (b) mobility trends; (c) health indicators; (d) demographics; (e) spatial factors and (f) temporal factors. The modeling approach will allow us to identify the factors that contribute to the overall hospitalization and ICU demand in US during the pandemic.

*Finally*, the model developed is employed to generate predictions (at various spatial units including country, region, state and county) for hospital and ICU usage for COVID and non-COVID patients under a host of future scenarios of COVID-19 transmission. The scenarios are generated considering potential influence of vaccination and the uncertainty associated with COVID-19 variants. The exercise provides an understanding of how hospitalizations and ICU bed usage rates might possibly vary over time across the country.

## Results

### Data collection and preparation

The primary focus of the analysis is to study factors affecting two measures: 1) hospitalization rates (measured as number of beds used) and 2) ICUs used. In our analysis, we examine these measures for COVID and non-COVID patients. Hence, a total of 4 dependent variables are analyzed including: COVID-19 hospitalization and ICU rate per 100 K population; and non-COVID hospitalization and ICU rate per 100 K population. The data for our analysis is drawn from the DHHS database compiled from approximately 5,000 hospitals encompassing 2,462 counties in the country^20^.The dataset provides information on weekly hospitalization and ICU data from July 31st, 2020 through January 22nd, 2021 (total 32 weeks data). For the analysis, we aggregate the weekly level hospital and ICU data at a county level and consider their natural logarithm to generate the final dependent variables.

In terms of independent variables, the following six broad categories are considered: COVID-19 transmission related factors; mobility trends; health indicators; demographics; spatial factors and temporal factors. An exhaustive list of these variables are presented in Table 1. A detailed description of the independent variables considered in the study is available in our earlier work^8^. Within the COVID-19 related factors, we consider different measures including county level weekly COVID-19 transmission rate, percentage change in the virus transmission rate compared to the preceding 3 week average, and an indicator variable for a possible increase in COVID cases in the last week. We collect the mobility trends from PlaceIQ^21^ dataset that provides daily exposure matrices through the smartphone movement data for counties with at least 100 devices active in a day. The exposure data is measured based on smartphone device exposure computed as the exposure of a device to distinct other devices at a specific point of interest. The exposure for all devices from a county are aggregated to generate a weekly average county level exposure metric^8,21^.

**Table 1:** Descriptive statistics of the dependent and independent variables.

Variables	Source	Mean	Min/Max	Sample size
**Dependent variables**				
Ln (COVID hospitalization rater per 100 k)	DPH^a^	1.935	0.000/7.525	37,065
Ln (non COVID hospitalization rater per 100 k)	DPH	4.069	0.000/8.341	37,065
Ln (COVID ICU rater per 100 k)	DPH	0.752	0.000/6.995	37,065
Ln (non-COVID ICU rater per 100 k)	DPH	1.536	0.000/6.752	37,065
**Independent variables**				
***COVID-19 related factors***				
COVID case per 100 people, 1 weeks lag	CSSE	5.008	0.000/4.560	37,065
COVID case per 100 people, 2 weeks lag	CSSE	5.008	0.000/4.560	37,065
difference from 3 week moving average	CSSE^b^	0.086	−1.000/2.002	37,065
Weekly COVID-19 cases higher than the moving average	CSSE	0.587	0.000/1.000	37,065
***Mobility trends***				
Ln (Daily Average Exposure), 2 weeks lag	CEI^c^	4.536	2.319/6.841	37,065
Ln (Daily Average Exposure), 3 weeks lag	CEI	4.521	2.324/6.841	37,065
***Demographic characteristics***				
Young people percentage	ACS^d^	22.403	7.155/35.987	1765
Hispanic percentage	ACS	10.015	0.653/96.322	1765
African American percentage	ACS	9.720	0.113/76.331	1765
Female percentage	ACS	50.348	37.041/56.145	1765
Ln (median income)	ACS	10.866	10.149/11.821	1765
Income inequality ratio (80th/20th percentile)	CHRR^e^	4.540	2.987/9.148	1765
***Health indicators***				
Asthma % for > = 18 years	CDC	9.417	7.400/12.300	1765
Ln (number of cardiovascular patients per 1000 Medicare beneficiaries)	CHRR	4.119	3.157/4.891	1765
Hepatitis C Cases per 100 K people	CDC^f^	1.064	0.000/5.600	1765
Ln (HIV rate per 100 K People)	CDC	4.780	0.723/7.859	1765
Ln (cancer rate per 100 K People)	CDC	6.119	5.489/6.436	1765
***Spatial factors***				
West region	USA map	0.120	0.000/1.000	1765
Mid-West region	USA map	0.108	0.000/1.000	1765
North-East region	USA map	0.308	0.000/1.000	1765
Top 10 tourist state	CHRR	0.252	0.000/1.000	1765
Number of airports per 100 k people	CHRR	1.269	0.000/24.927	1765

^a^Department of Health and Human services^20^; ^b^Center for Systems Science and Engineering Coronavirus Resource Center at Johns Hopkins University^22^; ^c^COVID Exposure Indices^21^; ^d^American Community Survey; ^e^County Health Rankings & Roadmaps; ^f^Central for Disease Control System.

For our analysis, we selected the 2,018 counties with mobility data available. Out of these 2,018 counties, there was a small sample of counties (~ 250) that do not have any hospitals and thus are excluded from our analysis. The final dataset consist of total 1,765 counties (out of 3,142 counties in US) with 21 weeks of data (from August 28th, 2020 to January 22nd, 2021) for each county. It is important to recognize that these 1,765 counties account for more than 95% of the US population and 97% of the reported COVID-19 cases.

**Figure 1:**
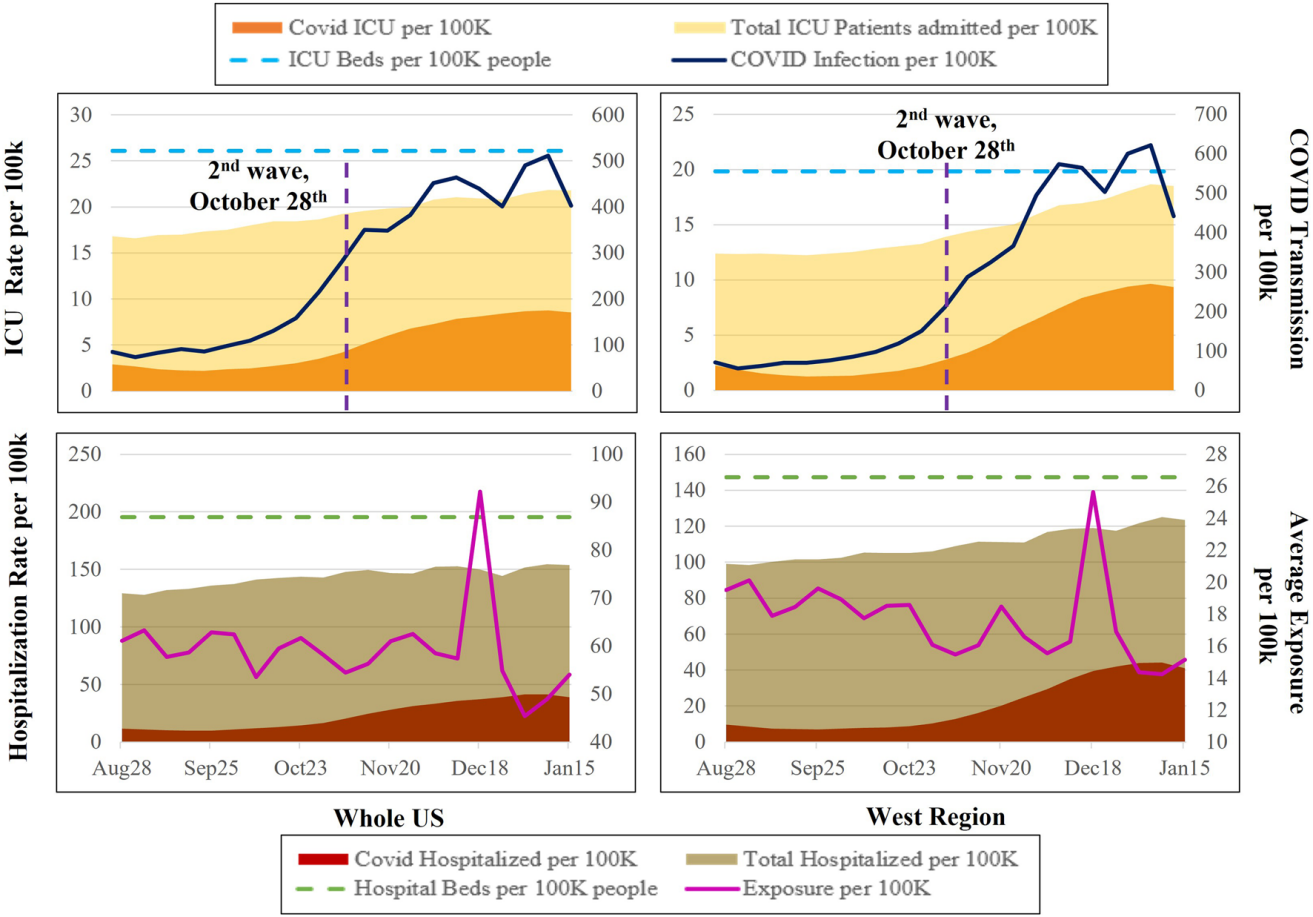
A representation of the hospitalization trends across the country and west region.

A visual representation of salient characteristics of the data are presented in Fig. 1. Specifically, we present four different measures including (a) hospitalization and ICU rate (total and by COVID patients), (b) COVID-19 transmission rates, and (c) average mobility exposure. To conserve on space, we restrict ourselves to presenting the measures for the US and west region. The corresponding statistics for Other regions’ are provided in the supplemental material, Figure A.1. From Fig. 1, we can observe how hospitals are inundated with COVID patients while the number of non-COVID patients are declining across the country. The situation is particularly worse in the West region as hospital beds and ICU units are occupied with the influx of COVID-19 patients. The ICU capacity in the west region dropped to 10% with an availability of only 3 ICU beds per 100 k people. All of these measures clearly depict the challenging situation impacting the hospital sector due to the pandemic. From the figure, we can also see that COVID 19 transmission rates continue to increase across the country with two surges in the month of November, 2020 and December, 2020. With respect to the mobility trends, the results indicate low mobility in the time periods August through November with a spike beginning in the middle of December that can be attributed to Christmas and New Year holiday associated gatherings.

### COVID-19 hospitalization model results

Table 2 provides COVID and non-COVID hospitalization model estimates. We restrict ourselves to a discussion of COVID hospitalization models. The discussion of the results for the non-COVID hospitalization rate (Section A.1), and estimates for ICU rate models (Table A.1) are included in the supplemental materials.

**Table 2:** Hospitalization model results.

Parameter	COVID Hospitalization		Non COVID Hospitalization	
	Estimate	t-statistics	Estimate	t-statistics
Intercept	−20.151	−11.971	−9.858	−6.025
**Covid-19 transmission factors**				
COVID case per 100 people, with 1 week lag	–	–	−0.076	−4.292
COVID case per 100 people, with 2 weeks lag	1.266	17.790	−0.107	−5.933
x Effect in the West Region	−0.145	−1.765	–	–
x Effect in the South Region	−0.694	−8.575	–	–
% difference with the preceding 3 week moving average	0.089	3.986	–	–
x Effect in the Mid−West Region*	0.177	5.894	–	–
x Effect in the South Region	−0.038	−1.653	–	–
Weekly COVID-19 cases higher than the moving average (base is covid-19 cases same or lower)	0.058	5.753	–	–
**Mobility trends**				
Ln (Daily Average Exposure) with a 2 weeks lag	0.157	7.285	–	–
Ln (Daily Average Exposure) with a 3 weeks lag	0.295	12.502	–	–
**Demographics**				
Young population percentage (19 years or less)	−0.051	−5.511	−0.043	−4.835
Hispanic percentage	0.025	11.141	0.008	3.426
African American percentage	0.020	9.431	0.002	0.844
Female percentage	0.173	10.719	0.153	9.758
Ln (median income)	0.619	5.250	–	–
**Health indicators**				
Ln (number of cardiovascular patients per 1000 Medicare beneficiaries)	1.133	9.215	–	–
Hepatitis C Cases per 100 K people	0.070	2.624	–	–
Ln (HIV rate per 100 K People)	–	–	0.236	6.307
Ln (cancer rate per 100 K People)	–	–	0.973	3.747
**Spatial factors**				
Region (Base: West, South, Pacific)				
Mid-West region			0.111	1.897
North East region	−0.109	−2.197	–	–
x Effect Since 2nd Wave started (October 30th)	0.204	3.923	–	–
Top10 tourist state	0.243	3.736	–	–
x Effect Since 2nd Wave started	−0.114	−3.061	–	–
Number of airports per 100 k people	0.017	1.651	–	–
**Temporal factors**				
Effect Since 2nd Wave started (October 30th)	0.360	18.212	–	–
Effect Since 25th December	0.106	6.422	−0.041	−3.786
**Correlations**				
$\sigma$^2^	1.912	49.925	1.370	40.445
$\rho$	0.930	453.710	0.965	791.200
$\Phi$	0.835	237.303	0.888	306.605

*The indented variable name presentation starting with “x” is adopted to indicate that the variable represents the interaction term with that specific variable.

*Intercept*: The intercept does not have any substantive interpretation after adding other independent variables.

*COVID-19 related factors*: The consideration of COVID-19 transmission rates in the model recognizes the delay of about 5 to 14 days between transmission and hospitalization. Hence, in our analysis we tested COVID-19 transmission variable with lag of 1, 2 and 3 weeks. Of these lag variables, the 2 weeks lag variable offered the best model fit. As expected, increase in COVID-19 transmissions is associated with increased hospitalization rate. Further, we find that the impact of transmission rates is lower (yet positive) in the West and South region relative to the rest of the country. In addition, in our analysis, to represent rising COVID-19 cases in the county, we defined several variables such as (a) an indicator variable for increasing cases defined as number of weekly cases greater than the 3 week moving average (3WMA) and (b) a percentage difference variable representing change in weekly cases relative to the 3WMA. These variable impacts follow expected trends. The indicator variable for increasing cases contributes to increasing hospitalization rates. The difference variable (can be positive or negative) indicates that hospitalizations are sensitive to percentage changes in COVID-19 transmission. The sensitivity is substantially higher for Mid-West region while being slightly lower for the South region.

*Mobility trends*: Consistent with earlier research, our analysis also highlights how increased mobility in the county results in higher hospitalization rates. We recognize that the impact of mobility on hospitalizations has a lagged effect. Hence, we tested weekly mobility with 2 weeks and 3 weeks lag. The two lag variables offer significant and intuitive results highlighting the role of mobility in addition to the impact of COVID-19 cases.

*Demographics*: With respect to demographic characteristics, we find that counties with higher percentage of young population (aged 19 years or less) are likely to have lower hospitalization rates^23^. The results for ethnicity composition variables also offer expected results. Counties with higher Hispanic and African American populations are likely to have higher hospitalizations, perhaps attributed to their residence in densely populated neighborhoods and pre-existing chronic medical conditions^23^. Interestingly, in our analysis, hospitalization rates are found to be higher for counties with higher female population. Finally, median county income is also positively associated with hospitalization rates, possibly manifesting the impact of access to hospitals in these counties.

*Health indicators*: Consistent with earlier findings, our analysis also shows a significant positive association between the COVID hospitalization rate and pre-existing health risk factors, especially for population with higher incidence of cardiovascular disease and hepatitis C^24^.

*Spatial factors*: In our analysis, we tested several spatial factors to account for inherent regional differences in hospital infrastructure, transportation infrastructure, tourism activity, weather patterns and regional culture. The results shows that north east region is likely to experience lower hospitalization rate due to COVID-19 compared to the rest of the country prior to October 30th. However, after October 30th, the north east region experienced slightly higher hospitalization rates. The higher hospitalization rate for this time period is closely aligned with the increasing caseloads across the country. Further, we considered the tourism status of the state in our analysis by identifying the top and bottom 10 desirable states with respect to tourism activity. As expected, we find a positive effect of the top 10 tourist attraction states on the COVID hospitalization rate, perhaps indicative of the higher virus transfer in these regions. However, the effect was marginally reduced from October 30th. Finally, the variable specific to the number of airports per 100 k people reveals a positive impact on the COVID hospitalization rate.

*Temporal factors*: With respect to temporal variables, we consider various functional forms including continuous (linear, square and other polynomial forms of week difference) and indicator variables (such as Pandemic effect since October 30th, 2020 (second wave), since mid of December and from 25th December, 2020 and later). As expected, we find a positive effect of the second wave variable indicating a higher hospitalization rate across the country from October 30th, 2020. The results also indicate that hospitalization rate for COVID patients accelerated further from 25th December, 2020.

*Correlation factors*: The last row panel of Table 2 present the correlation parameters (${\sigma }^{2}, \rho$ and $\phi$). The significant effect of the parameters clearly highlight the presence of correlation affecting the county COVID-19 hospitalization rate across multiple observations.

## Discussion

### Hospital demand projection

Prior to hospital demand projection into the future, we also evaluate the predictive performance of our proposed model in predicting all four response variable in context, to illustrate the applicability of our model. The figures are available upon request from the authors. The main objective of the current study is to contribute to public health planning by evaluating how hospitalizations trends evolve over time. Specifically, the focus is on drawing insights on future demand while accommodating for the inherent uncertainty in future COVID-19 transmission. In terms of future COVID-19 transmissions, the reader would note that while COVID-19 virus transmissions are receding currently, the emergence of highly transmissible variants might affect the trajectory. Towards this end, we consider four potential COVID-19 evolution scenarios as follows^25^:

1. *Peak and valley *(*PV*): In this scenario, COVID-19 cases are assumed to follow a series of repetitive COVID-19 waves (ups and downs) throughout the spring of 2021 and beyond. The waves could be a result of delays in vaccination production and/or vaccination drives resulting in a longer timeframe for COVID-19 cases to reduce.
2. *Unexpected third spike *(*UP*): In the presence of new variants of COVID-19, a potential spike in COVID-19 transmissions that might occur in March or April is considered in this scenario. In this scenario, while the spike results in increased transmission, active vaccination drive can contribute to case reduction starting from the end of April.
3. *Slow burn *(*SB*): In this scenario, we assume that the cases will go down slowly in the coming months while not diminishing completely until the end of July.
4. *Rapid vaccination *(*RV*): It is possible that multiple vaccine candidates might be approved (such as Johnson and Johnson and others) in the near future rapidly increasing US vaccination rates. In this scenario, COVID-19 transmission rates are likely to reduce and reach a very manageable level early summer.

**Figure 2:**
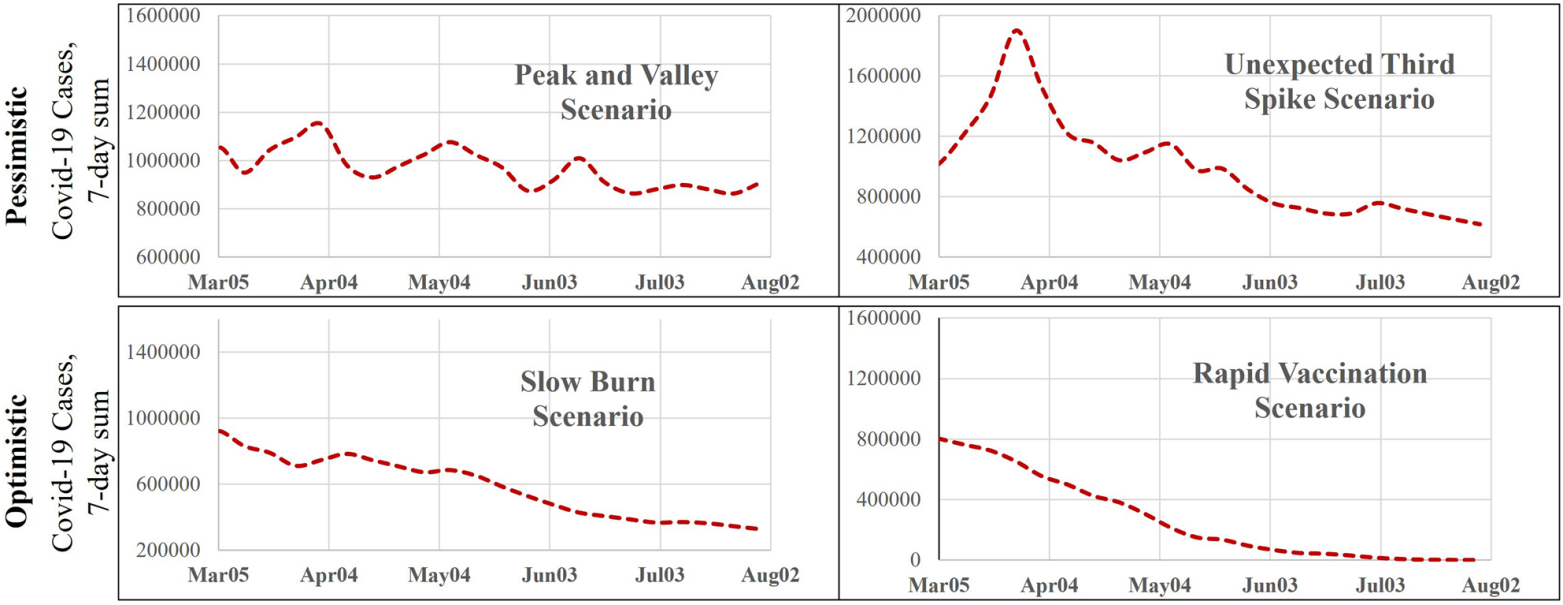
A representation of the assumed scenarios of the COVID-19 transmission rate in future.

The first two scenarios represent a pessimistic outlook towards COVID-19 transmission while the scenarios 3 and 4 represent an optimistic outlook. Figure 2 provides a representation of future COVID-19 transmission for the four scenarios described. The scenarios are generated employing percentage changes in cases at a county level thus ensuring spatial variability in the country. The spectrum of scenarios will enable us to examine the variation in hospital bed and ICU demand in the future. The reader would note here that mobility trends will also influence COVID-19 hospitalization demand. However, to focus on COVID-19 transmission trends, we consider the same mobility profile across all four scenarios (Figure A.2).

For these four scenarios, we forecasted hospital and ICU capacity using the proposed linear mixed model for all counties considered in the analysis over the next 22 weeks (March 5th to August 6th). The model predictions at the county level can be appropriately aggregated to any spatial resolution to provide insights at the national, regional, state and county level.

**Figure 3:**
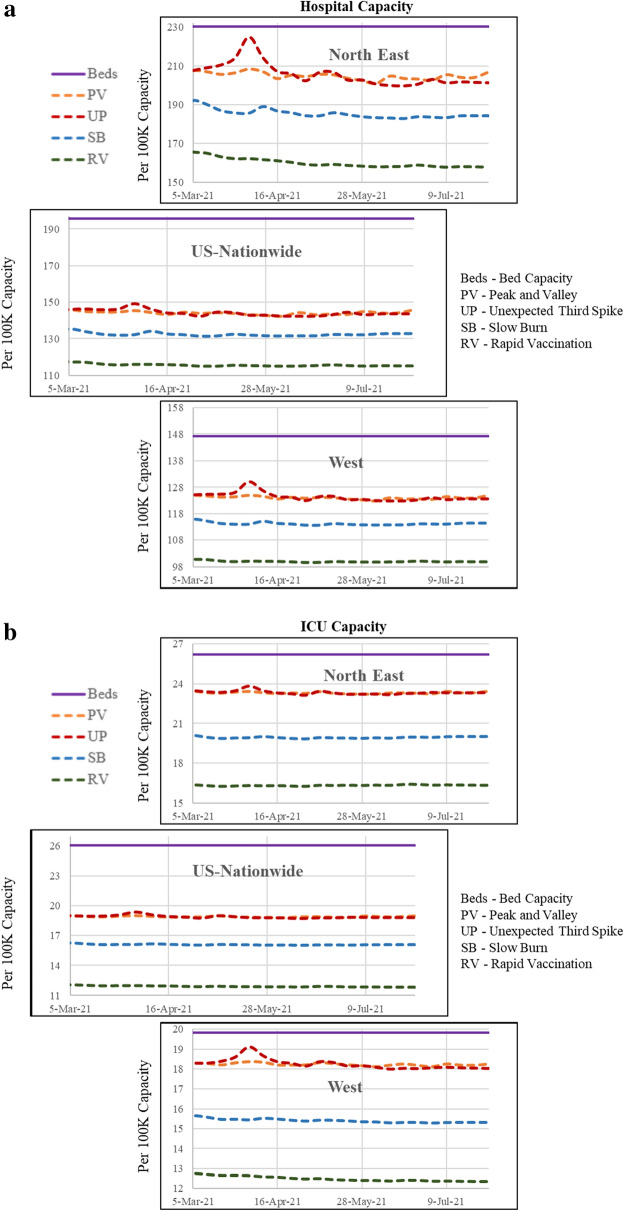
(**a**) Future hospital capacity across the country and regions (west and north-east) based on the hypothetical scenarios. (**b**) Future ICU capacity across the country and regions (west and north-east) based on the hypothetical scenarios.

A national and regional outlook for hospitalization and ICU rates are provided in Fig. 3a and b for the scenarios. In this set of figures, we present results for US, North-East and West regions for ease of presentation (results for other regions are presented in supplemental material—Figures A.3, and A.4). The results for all the scenarios follow expected trends—with the pessimistic scenarios showing higher demand and optimistic scenarios presenting with lower demand. The national results also clearly indicate that national hospital supply can meet the demand under all scenarios. However, it is important to recognize that excess demand in a county/region cannot be transferred to a county/region with deficit. An examination of the hospitalization and ICU rates in North-East and West reinforces this point. Under pessimistic scenarios, capacity in the North-East and West regions might come near the maximum available capacity (≥ 90%). Specifically, North-East region might experience a supply–demand mismatch for hospital beds while ICU supply–demand mismatch is expected to be more likely in the West region. These results reflect inherent regional and demographic differences across the country.

**Figure 4:**
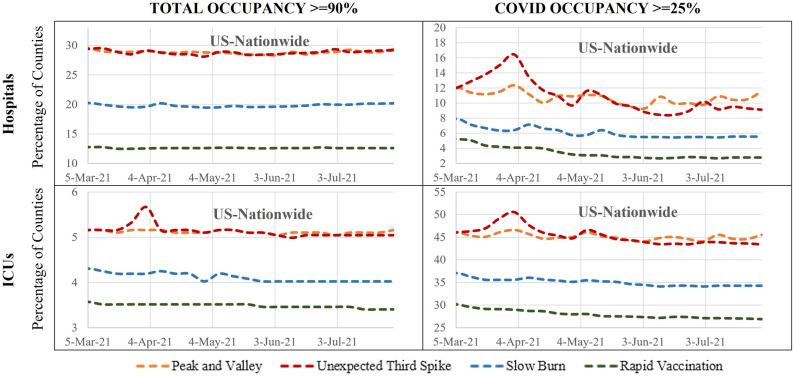
Number of counties with capacity over 90% and COVID patients over 25%.

To further understand the potential mismatch in demand and supply, we examine what percentage of the counties (out of 1,765) might potentially experience a supply and demand mismatch—defined as at least 90% hospital beds and ICU units being used. We also identify the percentage of counties with at least 25% of hospital beds or ICU units allocated to COVID-19 patients for the four scenarios. The results of this exercise, presented in Fig. 4, indicate that hospitalization rates exceed 90% in a range between 13–30% while for ICU usage, the rate vary from 3.5–5.5% of counties across the four scenarios. The number of counties with more than 25% COVID-19 hospitalizations (3–16%) or ICU usage (27–50%) varies significantly across the scenarios.

**Figure 5:**
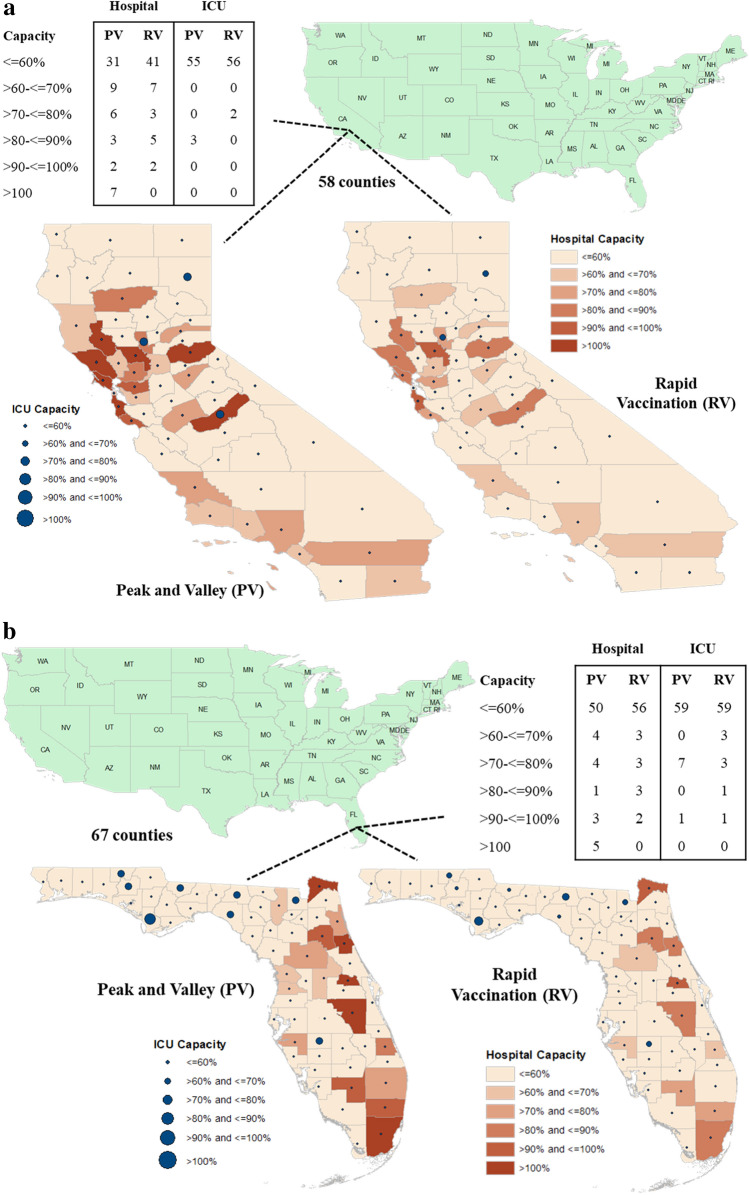
(**a**) Future hospital capacity at counties (California) based on the hypothetical scenarios. (**b**) Future hospital capacity at counties (Florida) based on the hypothetical scenarios.

The results presented so far examine the trends over time. To illustrate our model applicability for county level analysis at a specific time point, we present the results of two scenarios—PV and RV—in July across the states of California and Florida in Fig. 5a and b. The figures provide the following measures: (a) county level hospitalization and ICU capacity usage, and (b) number of counties within each category. As expected, higher number of counties are at risk of exceeding capacity in the PV scenario compared to the RV scenario in both states. The figures show how employing the proposed model system, counties at risk at any future time point can be identified to suggest remedial measures such as increasing the staff resources and/or hospital resources as needed. A state by state analysis of hospital capacity usage is presented in Figure A.5.

In summary, the framework proposed for understanding and quantifying hospitalization rate can allow policy makers to (a) evaluate the impact of COVID-19 virus transmission on hospitalization and ICU rates while controlling for demographics, health indicators and mobility trends so as to identify vulnerable counties that need to prioritized for vaccination; (b) estimate the hospitalization and ICU rates at the county, state level to identify COVID-19 transmission trends under a host of future scenarios (c) Identify vulnerable locations with low projected hospital and ICU capacity (County/State/Region) in advance so that remedial measures can be adopted to provide additional hospital infrastructure and access to personnel and (d) Develop a comprehensive plan for assisting hospital systems across the country to address displaced demand due to COVID-19.

To be sure, the paper is not without limitations. The data on the hospitalization rate are continuously updated for few counties to correct for errors or omission. Further, the hospitalization data used in the analysis is an administrative dataset obtained from DHHS and could possibly have over-estimated COVID-19 related hospitalization. A potential avenue for future research would be to reconcile the DHHS data with other hospitalization related datasets and develop updated models with the reconciled dataset. In the scenario analysis, we assume future mobility to be similar across the scenarios while it is quite possible that mobility might also be affected as virus transmission rates change. Therefore, for future research, it might be interesting to explore how the demand on the health care system varies based on mobility behavior. The proposed model is intended to serve as skeletal framework that can be readily updated with newer data on virus transmission and mobility patterns. The plots generated can be readily customized with more up to date information on COVID-19 cases to arrive at estimates of hospitalization and ICU demand. Finally, while we considered counties that account for 97% of the reported COVID cases, it is possible that counties not considered might contribute in small numbers to hospitalizations in the counties considered. Future research might address this limitation by developing spatial models that consider such spillover effects (from neighboring counties) in the analysis. Finally, with the growing availability of vaccination data, it would be useful to update the model using county level vaccination data in modeling hospitalization and ICU demand.

## Method

All the response variables considered in the study are continuous in nature and thus, a linear regression framework would be an appropriate choice for analyzing the data. However, in our data, every county is considered 21 times (21 weeks of data) and a simple linear regression method is not appropriate for such repeated measures^8^. Therefore we adopted a linear mixed approach (Autoregressive moving average -ARMA model structure, see^8^ for details) to accommodate for the influence of repeated observations at a county. A brief description of the linear mixed model is provided below:

Let *z* = 1, 2, … , Z = 1,725 be an index to represent county, *t* = 1, 2, … 21 be index to represent the week for each county. The equation for the linear mixed model can be written as:

1 $${y}_{zt}= \beta {X}_{zt} + {\varepsilon }_{zt}$$

where, ${y}_{zt}$ is the dependent variable (we have four dependent variables in the current study as stated in the data preparation section); $x$ is the vector of attributes and $\beta$ is the model coefficients. The random error term ${\varepsilon }_{zt}$, is assumed to be normally distributed across the dataset. To account for the repeated covariance measure, we used the ARMA structure. The exact functional form of the covariance structure assumed is shown below:

2 $$\Omega ={\sigma }^{2}\left(\begin{array}{cccc}1& \phi \rho & \dots & \phi {\rho }^{t-1}\\ \phi \rho & 1& \dots & \phi {\rho }^{t-2}\\ \vdots & \vdots & \ddots & \vdots \\ \phi {\rho }^{t-1}& \phi {\rho }^{t-2}& \dots & 1\end{array}\right)$$

where, ${\sigma }^{2}$ represents the error variance of $\varepsilon$, $\phi$ represents the common correlation factor across time periods, and $\rho$ represents the dampening parameter that reduces the correlation over time^26^. The models are estimated in SPSS using the Restricted Maximum Likelihood Approach (REML). The REML approach estimates the parameters by computing the likelihood function on a transformed dataset. The approach is commonly used for linear mixed models^27^.

## Supplementary Information

Supplementary Information.

## Data Availability

The datasets generated during and/or analyzed during the current study are available in the Department of Health and Human services repository, https://healthdata.gov/Hospital/COVID-19-Reported-Patient-Impact-and-Hospital-Capa/anag-cw7u.
